# Platelet dysfunction and inhibition of multiple electrode platelet aggregometry caused by penicillin

**DOI:** 10.1186/1477-9560-8-13

**Published:** 2010-07-21

**Authors:** Christian Schulz, Olga von Beckerath, Rainer Okrojek, Nicolas von Beckerath, Steffen Massberg

**Affiliations:** 1 Deutsches Herzzentrum and 1. Medizinische Klinik, Klinikum rechts der Isar, Technische Universität München, Munich, Germany

## Abstract

Beta-lactam antibiotics, e.g. penicillin, may inhibit platelet function and lead to reduced response in light transmission aggregometry and adhesion. However, influence on platelet function tests more commonly used in clinical practice, such as multiple electrode platelet aggregometry (MEA), have not been described so far. We report a case of a patient with local streptococcus infection. Treatment with penicillin resulted in mild bleeding tendency after 3 days. While coagulation parameters were normal, assessment of platelet function by MEA revealed strong platelet inhibition of both ADP and arachidonic acid induced platelet aggregation comparable to normal responders to antiplatelet therapy. Change of antibiotic regime resulted in recovery of platelet function. Thus, penicillin therapy may impact on platelet function and consecutively commonly used platelet function assays, e.g. MEA.

## Case report

Inhibition of platelet function in the presence of beta-lactam antibiotics was first described decades ago. In the first documented report in 1973, Cazenave et al. found that beta-lactam antibiotics (BLA), e.g. penicillin, inhibit all major types of platelet functions in vitro, such as secretion, adhesion and also aggregation [1]. Postulated mechanisms are inhibition of agonist-stimulated platelet calcium influx [2], and impairment of agonist binding to their specific receptors on the platelet surface [3]. Importantly, penicillin binds irreversibly to platelets [4]. This results in a dose- and time-dependent effect of BLA on platelet function in animals and humans, which occurs within 24 to 72 hours after therapy induction [4-6].

A 46-year old female patient presented to our clinic with a perimandibular abscess following surgical removal of a third molar. The patient had no medical therapy upon admission. Despite elevated inflammation parameters other blood tests were normal. The patient was treated with surgical drainage and intravenous penicillin (12 million IU/day) because of local infection with penicillin-sensitive streptococcus species. Due to swelling of the neck and jaw the patient required mechanical ventilation for three days. In addition to antibiotic therapy, the patient received fentanyl and midazolam intravenously for analgo-sedation, and enteral feeding by tube.

**Figure 1:**
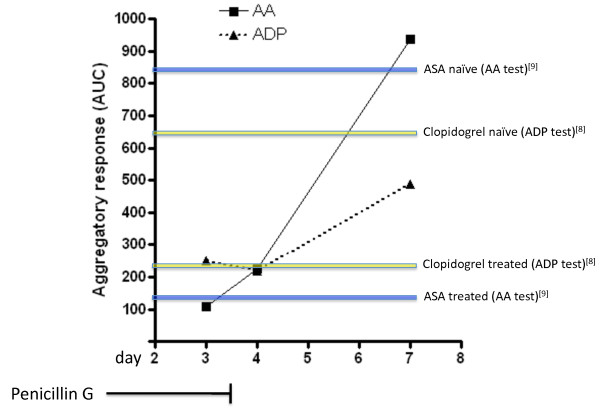
**Trend in platelet function in relation to penicillin therapy**. Platelet function was assessed by multiple electrode platelet aggregometry (AUC) in response to adenosine diphosphate (ADP) and arachidonic acid (AA). Intravenous penicillin therapy (12 million IU/day) was discontinued after 3 days of treatment. Colored lines indicate AUC mean values from human subjects in previous studies in response to either ADP (yellow) at baseline (naïve) and after receiving 600 mg clopidogrel [8], or AA (blue) before and after application of 500 mg acetylsalicylic acid (ASA) [9]. In the presence of penicillin, aggregatory response to ADP and AA was inhibited and MEA values were similar to normal responders to antiplatelet therapy.

On postoperative day 3 bleeding tendency during invasive procedure (routine placement of arterial catheter) was noticed. Platelet counts (174000 [150000-450000]/μl), partial thromboplastin time (PTT 31 [25-39] sec) and international normalized ratio (INR 1.0) were normal. However, multiple electrode platelet aggregometry (MEA)[7] revealed significant inhibition in platelet function in both adenosine diphosphate (ADP) and arachidonic acid (AA) tests (fig. 1). In detail, on day 3 MEA area under the curve (AUC) in response to ADP was 250, and 109 in response to AA respectively. In a recent study, MEA results from untreated and clopidogrel naïve subjects were 650.5 AUC [523.0-807.0] at baseline, and application of 600 mg of clopidogrel resulted in a significant reduction to 233.5 AUC [145.0-429.5][8]. Likewise, intravenous application of 500 mg acetylsalicylic acid resulted in mean values of arachidonic acid-induced aggregation (ASPI test) of 137 ± 44 [9]. Thus, the here presented inhibition of ADP- and AA-induced platelet aggregation in the presence of BLA is similar to patients receiving antiplatelet therapy (fig. 1).

Next, the antibiotic regime was changed to clindamycin and ciprofloxacin. After cessation of penicillin treatment, platelet MEA response increased (fig. 1). However, ADP-induced platelet aggregation was still abnormal 4 days after application of the last penicillin dose. Thus, penicillin treatment may lead to significant platelet inhibition, and normalization of platelet function is prolonged due to irreversible binding of penicillin to platelets [4]. Importantly, recovery of platelet function after discontinuation of treatment in vivo normally requires 3 to 7 days [6]. However, cases with abnormal aggregation for more than 2 weeks after cessation of penicillin therapy have been described [6].

In the era of coronary stent placement platelet function assays are widely used to assess platelet function under antiplatelet therapy. MEA is a common test to determine platelet reactivity under treatment with clopidogrel and acetylsalicylic acid, and results closely correlate with light transmission aggregometry [8]. MEA seems to be superior to other platelet function tests, e.g. VASP assay, in the prediction of adverse events such as stent thrombosis [10].

However, patients undergoing percutaneous coronary intervention (PCI) may develop infections of any kind that require medical treatment. Antibiotics are widely prescribed drugs for both in- and outpatient use [11,12]. It is, therefore, tempting to speculate that simultaneous application of antiplatelet drugs and beta-lactam antibiotics leads to significant variations in MEA values. In worst-case scenario, insufficient response to antiplatelet drugs, e.g. clopidogrel, may be disguised by the effect of BLA. Whether a combined treatment with beta-lactam antibiotics and antiplatelet drugs impacts on hemostasis, and whether the validity of commonly used platelet reactivity tests is influenced by BLA should be clarified by future studies. Also, it should be addressed if antiplatelet therapy in individuals with penicillin as a co-medication leads to increased bleeding events, and if in these patients antiplatelet therapy needs to be (temporarily) reduced.

While impairment of platelet function in vivo seems to be a rather common phenomenon in individuals receiving penicillin and also other BLA [4-6,13], occurrence of bleeding secondary to platelet dysfunction may vary [13]. These differences can be attributed to dose-dependent effects of penicillin and a consequence of irreversible binding to platelets [4-6], but also to the type of BLA chosen for treatment [13]. Further, blood serum constituents, e.g. albumin, may influence occurrence of bleeding in patients receiving BLA [14].

In summary, the here described case report suggests that patients under BLA therapy may display disturbed platelet function. As in our case, platelet inhibition can result in a significantly reduced aggregatory response to ADP and AA comparable to normal responders to antiplatelet therapy [15]. Therefore, in some cases it may be indicated to re-evaluate platelet function after discontinuation of BLA treatment.

## Consent

Written informed consent was obtained from the patient for publication of this case report. A copy of the written consent is available for review by the Editor-in-Chief of this journal.

## Competing interests

The authors declare that they have no competing interests.

## Authors' contributions

CS designed the study, analyzed data and wrote the manuscript. OvB designed the study and carried out platelet function tests. RO analyzed data. NvB designed the study. SM wrote the manuscript. All authors read and approved the final manuscript.
